# Treatment of *Neisseria meningitidis* prosthetic joint infections with short-course antibiotics: three cases and a literature review

**DOI:** 10.5194/jbji-6-33-2020

**Published:** 2020-09-04

**Authors:** Sarah Browning, Harry N. Walker, Thomas Ryan, Robert Pickles, Michael Loftus, Jason A. Trubiano, Andrew Hardidge, Joshua S. Davis

**Affiliations:** 1 Department of Infectious Diseases, John Hunter Hospital, Newcastle, NSW, Australia; 2 School of Medicine and Public Health, University of Newcastle, Callaghan, NSW, Australia; 3 Department of Infectious Diseases, Austin Health, Heidelberg, VIC, Australia; 4 Department of Orthopaedic Surgery, John Hunter Hospital, Newcastle, NSW, Australia; 5 Department of Infectious Diseases, The Alfred Hospital and Central Clinical School, Monash University, Melbourne, VIC, Australia; 6 Department of Medicine (Austin Health), University of Melbourne, Heidelberg, VIC, Australia; 7 Department of Infectious Diseases and The National Centre for Infections in Cancer (NCIC), Peter MacCallum Cancer Centre, Parkville, VIC, Australia; 8 Department of Orthopaedic Surgery, Austin Health, Heidelberg, VIC, Australia; 9 Global and Tropical Health Division, Menzies School of Health Research, Darwin, NT, Australia

## Abstract

*Neisseria meningitidis* is a rare cause of prosthetic joint infection (PJI), with only three cases previously reported. Here we report three further cases, all of which were successfully treated with implant retention and short-course antibiotics (<6 weeks).

## 1 Introduction

*Neisseria meningitidis* is a Gram-negative, aerobic, oxidase-positive diplococcus, for which humans are the only natural host. The organism is best known as a cause of purpura fulminans with bacteraemia and meningitis; however, urogenital infections, pneumonia, chronic bacteraemia, and native joint infections also occur (Murray et al., 2016; Barahona et al., 2017). Whilst virulent, *N. meningitidis* is typically very sensitive to beta-lactam antibiotics and fluoroquinolones, with successful outcomes using short courses of antimicrobials in the absence of surgical intervention reported in native joint septic arthritis (Cabellos et al., 2012). Similarly, it has long been recognised that the curative treatment of meningococcal ventriculitis, for example, in the context of a cerebrospinal fluid ventricular shunt does not necessitate removal of the device (Anderson and Yogev, 2005). Therefore, we hypothesised that meningococcal prosthetic joint infection (PJI) could be safely managed with minimal surgical intervention and short-course antibiotics, in contrast to more common PJI pathogens such as *Staphylococcus aureus*.

To our knowledge, there are only three previously reported cases of meningococcal prosthetic joint infection (Vikram et al., 2001; Becerril Carral et al., 2017; Mc Carthy et al., 2020). These cases were managed with at least 12 weeks of antimicrobial therapy, commencing with an intravenous beta-lactam followed by oral ciprofloxacin or amoxicillin (Table 1). We present three further cases of late acute haematogenous PJI due to *N. meningitidis*, demonstrating successful treatment with 6 weeks or less of antimicrobial therapy and a range of surgical interventions involving implant retention from single-needle arthrocentesis to multiple arthrotomies.

## 2 Case presentations

### 2.1 Case 1

A 70-year old woman presented 6 months following an elective right total knee arthroplasty (TKA) with 48 h of fever, rigors, nausea, and vomiting and 24 h of severe right knee pain and swelling. The right knee was warm and erythematous, with an effusion and reduced range of motion (ROM). Serum C-reactive protein (CRP) was 451 $\mathrm{mg}\;L^{-1}$ (<6.0 $\mathrm{mg}\;L^{-1}$), with neutrophilia of $13.9\times 10^{9}$ $L^{-1}$ (2.0–$8.0\times 10^{9}$ $L^{-1}$). Blood cultures taken at presentation showed no growth after 5 d of incubation.

Empiric treatment with intravenous piperacillin-tazobactam was commenced and rationalised to intravenous flucloxacillin (an anti-staphylococcal penicillin) 2 g 6-hourly after 24 h. On day 2 of admission 50 mL of purulent fluid was aspirated from the right knee under ultrasound guidance with $36\;000\times 10^{6}$ $L^{-1}$ leukocytes (95 % polymorphonuclear) and no organisms or crystals seen on microscopy. Synovial fluid culture was negative after 5 d of incubation.

Rapid clinical improvement was demonstrated, and she was able to mobilise independently with pain well controlled by the third day of admission. No surgical intervention was performed, and she was discharged home with a plan for 4 weeks of intravenous flucloxacillin via outpatient parenteral antimicrobial therapy for the treatment of probable PJI.

The result of 16s rRNA gene sequence analysis, performed on the initial synovial fluid specimen, was available following 3 weeks of intravenous flucloxacillin and detected genetic material demonstrating a 100 % match with *N. meningitidis*. Remel meningococcus agglutination serum test identified the meningococcus as serogroup W. Treatment was changed to ceftriaxone 2 g daily for a further 7 d, with no relapse of infection 5 years post treatment cessation. She achieved an excellent functional outcome. Antimicrobial prophylaxis was not recommended by public health in this setting given the prolonged period between patient contact and diagnosis.

### 2.2 Case 2

An 83-year old woman presented with 24 h of fever, headache, vomiting, diarrhoea and a painful swollen right knee on a background of elective right TKA 3 years earlier for osteoarthritis. She had a warm, swollen, irritable right knee with reduced ROM and no skin rash or meningism. Initial serum CRP was elevated at 72 $\mathrm{mg}\;L^{-1}$ (<6 $\mathrm{mg}\;L^{-1}$), with neutrophilia of $10.2\times 10^{9}$ $L^{-1}$ (2.0–$8.0\times 10^{9}$ $L^{-1}$). A plain X-ray of her right knee showed no signs of component loosening. Synovial white blood cell count was $>100\;000\times 10^{6}$ $L^{-1}$ (100 % polymorphonuclear leukocytes).

*N. meningitidis* was cultured from blood and synovial fluid samples with identification of colonies using VITEK MS MALDI-TOF (database V3.0 KB-clinical use), while bioMerieux Etest method and Clinical Laboratory Standards Institute (CLSI) interpretive criteria demonstrated susceptibility to ceftriaxone, ciprofloxacin, and rifampicin. Remel meningococcus agglutination serum test identified the meningococcus as serogroup W. The patient's husband was identified as a close contact and prescribed prophylaxis with oral ciprofloxacin in the community.

Empiric intravenous flucloxacillin, gentamicin, and ciprofloxacin were rationalised to intravenous ceftriaxone 2 g twice daily on day 2, as meningitis had not been ruled out, and lumbar puncture was contraindicated due to anticoagulation. Operative open right knee washout for debridement and implant retention (DAIR), including polyethylene liner exchange and extensive synovectomy, was performed on day 2. Copious pus was present within the joint capsule. There was no growth on tissue, swab, or sonication specimens.

Rapid clinical and biochemical improvement was noted post-operatively. She received 7 d of intravenous ceftriaxone 2 g twice daily, followed by a further 14 d of oral ciprofloxacin 750 mg twice daily. At 20 months following surgical debridement, her functional state was at premorbid level, with no clinical signs or symptoms of relapse.

**Table 1:** Summary of meningococcal prosthetic joint infection case reports.

Case no. (ref.)	Patient details	Diagnostic test	Operative management	Antimicrobial management
1 (this report)	70 F, 6 months post index R TKA	*N. meningitidis* (serogroup W) identified in synovial fluid using 16s rRNA gene sequence analysis	No surgical intervention performed	3 weeks IV flucloxacillin 8 $g\;d^{-1}$ continuous infusion, followed by 7 d IV ceftriaxone 2 g OD (4 weeks total therapy)
2 (this report)	83 F, 3 years post index R TKA	*N. meningitidis* (serogroup W) cultured from blood and synovial fluid	DAIR including polyethylene liner exchange and extensive synovectomy	7 d IV ceftriaxone 2 g BD, followed by 14 d PO ciprofloxacin 750 mg BD (3 weeks total therapy)
3 (this report)	75 M, 3 years post index L TKA. Polymicrobial infection involving L TKA, R hip and L wrist	*N. meningitidis* (serotype Y) cultured from blood	Open washout days 3 and 8. DAIR and polyethylene liner exchange day 10	3 d IV ceftriaxone 2 g OD, followed by 12 d IV benzylpenicillin 10.8 $g\;d^{-1}$ followed by 4 weeks PO ciprofloxacin 750 mg BD (6 weeks total therapy)
4 (Vikram et al., 2001)	80 F, 3 years post index R TKA	*N. meningitidis* (serogroup Y) cultured from synovial fluid	Open surgical drainage with implant retention	6 weeks IV ceftriaxone (dose unspecified), followed by indefinite PO penicillin V (dose unspecified)(indefinite course)
5 (Becerril Carral et al., 2017)	78 F, 7 months post index L TKA	*N. meningitidis* (serogroup B) cultured from synovial fluid	Arthroscopic debridement with implant retention	3 weeks IV ceftriaxone 2 g OD, followed by 9 weeks PO ciprofloxacin 750 mg BD (12 weeks total therapy)
6 (Mc Carthy et al., 2020)	72 F, 7 years post index L TKA	*N. meningitidis* cultured from synovial fluid and Gram-negative diplococci on Gram stain of blood cultures	DAIR and polyethylene tibial insert exchange	6 weeks IV ceftriaxone 2 g OD, followed by 6 weeks PO amoxicillin (dose unspecified) (12 weeks total therapy)

Abbreviations: IV: intravenous; F: female; M: male; TKA: total knee arthroplasty; R: right; L: left; DAIR: debridement and implant retention; OD: once daily; BD: twice daily; PO: per oral

### 2.3 Case 3

A 75-year old man presented with 4 d of fevers, rigors, myalgia, and lethargy following a 1 d history of pharyngeal pain. Past medical history included an elective left TKA performed 3 years prior for osteoarthritis. No focal infective features were initially noted, and empiric treatment with flucloxacillin was commenced pending blood culture results. Repeat physical examination in the first 24 h of admission revealed pain and reduced range of motion in the right hip, left wrist, and left knee. There was no rash at any time.

Needle aspirates of synovial fluid from the affected native right hip and left wrist performed on day 1 of admission revealed leukocyte counts of $468\;000\times 10^{6}$ $L^{-1}$ and $126\;000\times 10^{6}$ $L^{-1}$, respectively. Gram-negative diplococci were seen on Gram stain of both samples with antibiotics subsequently changed to intravenous ceftriaxone 2 g daily. The symptomatic left prosthetic knee joint was not aspirated.

*N. meningitidis* serotype Y was grown from blood within 24 h of collection (VITEK MS MALDI-TOF). Minimum inhibitory concentration was 0.064 $\mathrm{mg}\;L^{-1}$ for penicillin and 0.004 $\mathrm{mg}\;L^{-1}$ for ciprofloxacin (Etest, BioMérieux Inc, Marcy L'Etoil, France). Ceftriaxone was ceased and intravenous benzylpenicillin 10.8 $g\;d^{-1}$ was started on day 4 once antimicrobial sensitivities were finalised.

Operative washout of all three affected joints was performed on day 3, at which time the total leukocyte count in synovial fluid from the left prosthetic knee joint was $86\;000\times 10^{6}$ $L^{-1}$, with no organisms seen on Gram stain and no growth from culture. Two further debridement and washout procedures were performed on the prosthetic knee joint on days 8 and 10 of admission. The polyethylene liner was replaced during the final procedure, with the implant retained. Intravenous benzylpenicillin was continued until day 12, followed by ciprofloxacin 750 mg orally 12-hourly to complete 6 weeks of total antibiotic therapy. There were no symptoms or signs of relapse at time of follow-up 24 months post cessation of antibiotics.

## 3 Discussion

To date, only three cases of meningococcal PJI have been reported (Table 1), all of which were treated with either open or arthroscopic debridement with the implant retained and at least 12 weeks of antibiotics. Native joint septic arthritis (SA) is also an unusual presentation of meningococcal infection, accounting for just 1.4 % or 4 of 278 reported cases of invasive meningococcal disease in one case series in 2018 (Lahra et al., 2020), typically involving the knee and preceded by upper respiratory tract infection in 25 %–50 % of cases (Mc Carthy et al., 2020). In Australia, 1.4 % of invasive meningococcal isolates were resistant to penicillin in 2018 (Lahra et al., 2020).

Data from small case series suggest that, when meningococcal SA complicates another invasive meningococcal disease syndrome, it may be safely treated with as few as 4 d of antibiotics. Cabellos et al. (2012) identified 15 cases of “probable” SA between 1977 and 2010, all of whom were treated with a 4 d course of targeted antimicrobial therapy with no surgical intervention. Cure was achieved in all cases, with excellent functional outcomes.

The treatment of a PJI typically requires combined surgical and medical management, with a goal of removal or reduction of the biofilm burden to allow effective penetration of post-operative antibiotic therapy (Tande et al., 2017). Following DAIR, the treatment of most pathogens typically necessitates a 12–24-week course of either specific intravenous or highly bioavailable oral antimicrobial therapy (Li et al., 2018). While rifampicin-containing regimens are recommended for staphylococcal PJIs due to an association with higher cure rates (Tande et al., 2017), rifampicin was not used in any of the cases presented, and there is no evidence to support its use for meningococcal PJI.

While the authors remain confident that case 1 represents a true case of *N. meningitidis* PJI, it is important to note that certain factors cast possible doubt, including a failure to culture the organism from joint fluid following administration of piperacillin-tazobactam for the preceding 24 h and the subsequent clinical improvement despite use of an anti-staphylococcal penicillin. The rapid clinical improvement in the three cases described, despite short-course antimicrobial therapy and the absence of surgical debridement in case 1, may reflect a lack of certain virulence factors amongst meningococci, such as the ability to form biofilm. Although capable of biofilm formation, isolates from cases of invasive meningococcal disease in experimental models were less likely to form biofilm when compared to carriage strains (12 % vs. 30 %) (Yi et al., 2004). The ability of a strain to form biofilm is related to capsular expression, with non-biofilm-forming strains tending to be encapsulated (Lappann and Vogel, 2010). Encapsulated strains are poorer long-term colonisers but appear to demonstrate higher transmission frequency and thus persist in the human population. A lack of biofilm formation may explain successful treatment with antibiotics alone for meningococcal joint infections.

Although these patients were all over the age of 65 years, none of them had received meningococcal vaccination as they did not meet current Australian indications given the absence of risk factors for invasive meningococcal disease.

## 4 Conclusion

The clinical literature describing native joint meningococcal SA demonstrates that cure can be achieved with short antibiotic durations, even in the absence of synovial fluid drainage. Microbiology literature demonstrates that *N. meningitidis* strains isolated from cases of invasive meningococcal disease are unlikely to form biofilm. The three cases described above demonstrate the potential for meningococcal PJI to be cured with short-course antimicrobial therapy (3 to 6 weeks in total) and minimal surgical intervention and suggests that the potential risks associated with repeated orthopaedic interventions and prolonged antibiotic therapy could be safely avoided in future cases.

## Data Availability

No data sets were used in this article.
